# Optimization of Pressurized Liquid Extraction and In Vitro Neuroprotective Evaluation of *Ammodaucus leucotrichus.* Untargeted Metabolomics Analysis by UHPLC-MS/MS

**DOI:** 10.3390/molecules26226951

**Published:** 2021-11-17

**Authors:** Norelhouda Abderrezag, Jose David Sánchez-Martínez, Ouahida Louaer, Abdeslam-Hassen Meniai, Jose A. Mendiola

**Affiliations:** 1 Laboratory of Environmental Process Engineering, Faculty of Process Engineering, University Salah Boubnider–Constantine 3, Constantine 25000, Algeria; houdaabderrezag@gmail.com (N.A.); wlouaer@yahoo.fr (O.L.); abdeslam.meniai@univ-constantine3.dz (A.-H.M.); 2 Foodomics Laboratory, Bioactivity and Food Analysis Department, Institute of Food Science Research CIAL (CSIC-UAM), C/Nicolás Cabrera 9, 28049 Madrid, Spain; jd.sanchez.martinez@csic.es

**Keywords:** *Ammodaucus leucotrichus*, bioprospecting, pressurized liquid extraction, UHPLC-q-TOF-MS/MS, neuroprotective potential, anti-inflammatory activity

## Abstract

*Ammodaucus leucotrichus* is a spontaneous plant endemic of the North African region. An efficient selective pressurized liquid extraction (PLE) method was optimized to concentrate neuroprotective extracts from *A. leucotrichus* fruits. Green solvents were tested, namely ethanol and water, within a range of temperatures between 40 to 180 °C. Total carbohydrates and total phenolics were measured in extracts, as well as in vitro antioxidant capacity (DPPH radical scavenging), anticholinesterase (AChE) and anti-inflammatory (LOX) activities. Metabolite profiling was carried out by ultra-high-performance liquid chromatography coupled to quadrupole time-of-flight tandem mass spectrometry (UHPLC-ESI-q-TOF-MS/MS), identifying 94 compounds. Multivariate analysis was performed to correlate composition with bioactivity. A remarkable effect of the temperature using water was observed: the higher temperature, the higher extraction yield, the higher total phenolic content, as well as the higher total carbohydrates content. The water extract obtained at 180 °C, 10.34 MPa and 10 min showed meaningful anti-inflammatory (IC50_LOX_ = 39.4 µg/mL) and neuroprotective activities (IC50_AChE_ = 55.6 µg/mL). The Principal Components Analysis (PCA) and the cluster analysis correlated these activities with the presence of carbohydrates and phenolic compounds.

## 1. Introduction

The bioprospecting of the North African plants, especially from desert areas, is almost nonexistent compared with those on other continents, although the desert has a rich heritage of medicinal plants of huge diversity. Furthermore, the ability of plant adaptation to the extreme conditions of desert climate led to the synthesis of new molecules, which possess a wide range of interesting biological activities [1].

*Ammodaucus leucotrichus* (AL) is a spontaneous endemic plant belonging to Apiaceae family that is native of the Saharan and sub-Saharan countries of north and tropical Africa [2]. Morphologically, it is a small yearly wild and cultivated plant from 10 to 12 cm tall with fine and little fleshy leaves and white flowers grouped in umbels of 2, until 4 branches with 5 free petals. The fruit is diachene 6 to 10 mm long and has a dense, soft and white hair [3,4]; in fact, it is known in some areas as *hairy cumin*. Traditionally, it has been used in infusions or decoctions to treat cardiac disease [5], digestive problems [1,2,6] diabetes [7,8], aphrodisiac and tonic [6], in addition to being used as condiment and flavoring agent in tea and food seasonings [4]. AL fruit extract is reported to exhibit antioxidant [1,9], antibacterial [1,10], anti-inflammatory [10] and neuroprotective activities [11]. Most of the previous studies about AL were related to the phytochemical composition and the pharmacological activity of essential oil [1,2,11]. However, very little information is available about the phenolic compounds and their bioactivity [10,12].

Nowadays, the research of green and efficient extraction methods to obtain bioactives is of utmost interest, due to the different challenges to protect the environment and to achieve Sustainable Development Goals proposed by United Nations. Pressurized liquid extraction (PLE) is one of these innovative extraction methods, which employs liquid solvents in particular conditions of temperature and pressure up to the critical point [13,14]. Therefore, the viscosity and the surface tension of solvent decrease, while the solubility, the kinetic of desorption and analyte diffusion rate are improved. This technique has attracted great attention, especially in the pharmaceutical and food industries, as it is a rapid, automatic, inexpensive and efficient technology with the ability to cover a wide range of compounds polarities using generally recognized as safe (GRAS) solvents, such as water (ε = 80), ethanol (ε =24) and limonene (ε =2.3) [13]. Previous studies have confirmed the efficiency of PLE for the isolation of bioactive compounds from different plant materials, such as polyphenols [15,16], terpenoids [17,18], proteins [19], carotenoids [20] and lipids [21,22].

Alzheimer’s disease (AD) is the most prevalent degenerative brain disease and progressive neurological disorder, whose symptoms gradually worsen over time, as well as their incidence. AD is a complex multifactorial disease, which has become one of critical unsolved medical issues. It affects approximately 45 million people around the world—a number expected to reach 80 million by 2050 [23]. AD is mainly characterized by cognitive dysfunctions, neuroinflammation and oxidative stress. Acetylcholine (ACh) is the principal neurotransmitter in the brain, and its reduction level has been related to cognitive dysfunctions [24]. For that reason, acetylcholinesterase (AchE) inhibitors are the main drugs that are currently used for the treatment of the dementia associated with AD, which prevent the ACh neurotransmission [25]. On the other hand, neurodegenerative diseases such as AD are frequently associated with inflammatory processes. Lipoxygenase enzyme (LOX) is related to the neuroinflammation [26], and may also be one of the key mediators in neurodegenerative disease. Several studies suggest that LOX inhibitors may provide new treatment opportunities for AD and other neurodegenerative diseases [27]. In this sense, previous works indicated that plants belonging to the Apiaceae family, like *Ammodaucus leucotrichus*, were capable of influencing the nervous system [11,28]. This information reinforces our plant choice.

The aims of this present work was to apply for the first time a green extraction technique, PLE, with green solvents, to obtain bioactive molecules from *Ammodaucus leucotrichus* and to quantify total phenolic content (TPC) and total carbohydrate content. Furthermore, the in vitro biological activities such as antioxidant (DPPH), anti-acetylcholinesterase (AChE) and anti-inflammatory (LOX) will be evaluated. Meanwhile, ultra-performance liquid chromatography-quadrupole time-of-flight tandem mass spectrometry (UHPLC-q-TOF-MS/MS) techniques were selected to determine chemical profile extracts.

## 2. Materials and Methods

### 2.1. Plant Material

Wild *Ammodaucus leucotrichus* Cross. Dur. were harvested in Adrar in March 2019, a desert area in the southwest of Algeria, at an altitude of 285 m above sea level. A cryogenic mill (Cryomill, Retsch, Haan, Germany) was used to ground the plant fruits, as shown in Figure S1. The average particle diameter was less than 0.5 mm. The granulated sample was stored at 4 °C, ready for use.

### 2.2. Chemicals

Acetylthiocholine iodide (ACth), Acetylcholinesterase (AChE) from *Electrophorus electricus* (electric eel, Type VI-S), Trizma base (2-amino-2-(hydroxymethyl)-1,3-propanediol), Butylated Hydroxytoluene (BHT), Linoleic acid (LA), Lipoxidase (LOX) from Glycine max (soybean) Type 1-B, Fluorescein sodium salt, 2,2-diphenyl-1-picrylhydrazyl (DPPH), quercetin, Gallic acid, D-(+)-Glucose and Phenol were purchased from Sigma Aldrich (Madrid, Spain). Galantamine hydrobromide was from TCI Chemicals (Tokyo Chemical Industry Co., Tokio, Japan). Sulfuric acid (H_2_SO_4_) and Dimethyl sulphoxide (DMSO) and solvents such as Methanol, Ethanol, Acetonitrile and formic acid, LC-MS grade, were obtained from VWR chemicals (Radnor, Pensilvania, USA). 7-Fluoro benzofurazan-4-sulfonamide (ABD-F) was obtained from Alfa Aesar (Kandel, Germany). Sodium carbonate was from AppliChem Panreac (Barcelona, Spain). Merck KGaA (Darmstadt, Germany) provided the Folin-Ciocalteu reagent. Sea sand (0.1–0.6mm diameter) was from LabKem (Barcelona, Spain). All the water solutions were prepared with ultrapure water obtained from the Milli-Q system supplied by Millipore (Billerica, MA, USA).

### 2.3. Pressurized Liquid Extraction (PLE)

The extractions were performed in an Accelerated Solvent Extractor (ASE 200, Dionex, Sunnyvale, CA, USA). Prior to its use for extraction, each solvent was degassed in an ultrasound bath to prevent oxidation. For all the experiments, 1 g of ground raw material was mixed with 4 g of sea sand (dispersive agent) in an 11 mL stainless-steel extraction cell with cellulose filters at both sides (to avoid the passage of suspended particles to the collection vials).

After preliminary experiments, the static extraction time was fixed at 10 min. The samples were extracted using green solvents (ethanol or water) at 10.34 MPa (1500 psi) and at different tested temperatures of 40, 110 and 180 °C to cover a wide range of dielectric constant [18]. All the experiments were carried out in triplicate. Samples extracted with ethanol were dried using nitrogen stream, while those extracted with water were freeze dried (Lyobeta, Telstar, Terrassa, Spain); the obtained extracts were protected from light and frozen to avoid degradation. The extraction yield was calculated according to the following expression:

Extraction yield (*w*/*w*) (%) = dry extract mass (g) × 100/dry initial mass (g) (1)

### 2.4. Extract Characterization

#### 2.4.1. Determination of Total Phenolic Compounds (TPC)

The total phenolic compound was performed using the Folin–Ciocalteu method as previously reported [29], but with some modifications. Briefly, 10 μL of extract solution (concentration of 10mg of extract/mL EtOH) was mixed with 600 μL of water milli-Q. Then, 50 µL undiluted Folin-Ciocalteu reagent was subsequently added. After 1 min, 150 µL of 20% (*w*/*v*) Na_2_CO_3_ was added and the volume was adjusted to 1 mL with water. The mixture was vortexed and incubated at room temperature for 2 h in dark conditions, and then 300 μL was transferred to a 96-well microplate. The absorbance was measured at 760 nm with a Synergy HT microplate reader, Bio-Tek instruments (Winooski, VT, USA). The calibration curve was established using 0.031–2 mg gallic acid/mL EtOH, and was used to calculate the TPC of extracts expressed as milligram gallic acid equivalents (mg GA/g extract).

#### 2.4.2. Determination of Total Carbohydrate (TC)

Total carbohydrate was determined according to the Phenol-sulfuric acid method described by Dubois et al. [30]. First, 278 μL of extract (diluted in Milli-Q water at known concentration) were mixed with 167 μL of 5% phenol. Then, 1 mL of concentrated sulfuric acid (H_2_SO_4_) was added, and the mixture was vortexed. After 30 min of incubation at room temperature, the mixture was transferred into a 96-well microplate and the absorbance was measured at 490 nm. The total carbohydrate was calculated from a calibration curve using glucose as standard (from 6.25 to 100 μg/mL). The data were expressed as mg carbohydrate/g extract.

#### 2.4.3. Liquid Chromatography-Tandem Mass Spectrometry (UHPLC-q-TOF-MS/MS)

An ultra-high performance liquid chromatography (UHPLC) system 1290 from Agilent (Santa Clara, CA, USA), coupled with a quadrupole time-of-flight mass spectrometry (QTOF-MS) Agilent 6540 equipped with an electrospray ionization (ESI) source operating in positive and negative mode was employed for the phytochemical profiling of *Ammadaucus leucotrichus* seeds extracts.

Separation was achieved on a Zorbax Eclipse Plus C18 column (2.1 × 100 mm, 1.8 μm from Agilent Technologies, Santa Clara, CA, USA). The mobile phase consisted of A (0.1% Formic acid in water) and B (0.1% Formic acid in acetonitrile). According to the following gradient: 0 min, 0% B; 7 min, 30% B; 9 min, 80% B; 11 min, 100% B; 13 min, 100% B; and 14 min, 0% B. The column temperature was held at 40 °C and the flow rate was 0.5 mL/min, while the injection volume was 2 μL.

The MS was operated in positive and negative ionization modes. Other parameters were: capillary voltage, 3 KV; nebulizer pressure, 40 psi; drying gas flow rate, 11 L/min; gas temperature, 300 °C; skimmer voltage, 45 V; fragmentor voltage, 110 V, scanning range, 25–1100 *m*/*z*. The internal mass calibration solution of the Q/TOF was composed by 2 compounds: 5 μM of purine ([C_5_H_5_N_4_]^+^ 121.050873 *m*/*z*) and 2.5 μM HP-0921, hex-S-5akis(1H,1H, 3H-tetra—fluoropropoxy) phosphazine ([C_18_H_19_O_6_N_3_P_3_F_24_]^+^ at 922.009798 *m*/*z*) in acetonitrile-water (95:5, *v/v*) from Agilent.

The Mass Hunter Workstation software 4.0 (Agilent, Santa Clara, CA, USA), from Agilent was used for data acquisition and processing. All the data were converted to the mzXML file format. The MS/MS data of all the samples of each mode were uploaded and processed by Global Natural Products Social Molecular Networking (GNPS) (https://gnps.ucsd.edu, accessed 3 September 2021) [31]. The parameters of GNPS were set to a cosine score of >0.7 with a minimum requirement of 6 ions to match, precursor mass tolerance of 0.02 Da and the fragment ion mass tolerance of 0.02 Da. Additionally, identification of detected compounds was verified, also using the following databases: PubChem; HMDB; PhytoHub; and/or Massbank. Semiquantitative analysis was done just for comparison purposes of detected compound area among extracts.

### 2.5. Bioactivity Tests

#### 2.5.1. DPPH Radical Scavenging Assay

Free radical scavenging capacity was determined using DPPH· radical according to the method of Brand-Williams et al. [32]. Each well was filled with different volumes from 10 μL to 100 μL of sample, starting with a concentration of 1.5 mg/mL, were mixed with 150 μL of DPPH solution (6 × 10^−5^ M in MeOH). After 30 min of reaction in the dark, at room temperature, the absorbance was measured at 517 nm by the above-mentioned microplate reader. The results were expressed as radical scavenging activity percentage of the DPPH. The effective concentration having 50% radical inhibition (IC50) was calculated by linear regression from a graph where the abscissa represented the extract concentration and the ordinate the free radical scavenging activity. Solvent (use to dissolve the samples) plus plant extract solution was used as a blank, while the mixture of DPPH solution (150 μL; 6 × 10^−5^ M) and solvent was used as a negative control. BHT was used as positive control. Results were expressed as IC50 in µg/mL, with concentration required to reduce the 50% of the initial DPPH concentration.

#### 2.5.2. AChE Assay

The inhibitory capacity of AChE was measured by the Ellman method, with modifications proposed by Sańchez-Martínez et al., which used a fluorescence-based methodology to overcome difficulties of colored extracts [33]. ACth (Acetylthiocholine iodide) was employed as a substrate of the reaction, while ABD-F (4-Fluoro-7-sulfarnoylbenzofurazan) was used for the measurement of the cholinesterase activity. Mixtures of 100 μL of extract sample at different concentrations (8.33 μg–500 μg/mL) in EtOH/H_2_O (1:1, *v/v*) for the extracts using EtOH or in DMSO-H_2_O (1:1, *v/v*) for those using H_2_O, 100 μL of buffer (150 mM Tris-HCl pH = 8) and 25 μL of 0.8 U/mL AChE in buffer. Incubation was performed for 10 min at room temperature. The reaction was initiated by the addition of 25 μL of ABD-F (125 μM) in buffer and 50 μL of ACth at calculated K_m_, which is the concentration of substrate needed to obtain half of the maximum enzyme reaction velocity. The fluorescence measurements were done at λ_excitation_ = 389 nm and λ_emission_ = 513 nm every minute for 15 min at 37 °C. The degree of inhibition (DI) was calculated using the following Equation (2)

DI (%) = V_0_ − V_i_/V_0_ (2)

where V_0_ and V_i_ are the mean velocity obtained for AChE in the absence and presence of the inhibitor, respectively. Galantamine was used as reference inhibitor. Results were expressed as IC50 in µg/mL, and concentration was required to reduce the 50% of the initial acetylcholinesterase activity.

#### 2.5.3. LOX Assay

The inhibitory capacity of LOX was determined as described by Whent et al. [34]. The samples extracted using EtOH were diluted in EtOH-H_2_O (0.25:0.75, *v/v*), while those extracted with H_2_O were diluted with DMSO-H_2_O (0.25:0.75, *v/v*) at concentrations between 7.142–714.28 μg/mL. In each well, a mixture consisted of 100 μL of extract at different concentrations, 75 μL of fluorescein (1 μM) in buffer (150 mM Tris-HCl pH 9), 75 μL of LOX 208 U μL−1 in buffer and LA (in a concentrate that corresponds to the KM value) in EtOH/H_2_O (0.25:0.75, *v/v*). The amount of fluorescence resulting from the inhibition of LOX was determined using 485 nm excitation wavelength and 530 nm emission wavelength conditions every minute for 15 min at 25 °C. The degree of inhibition of LOX (DI) was calculated using Equation (2). Quercetin was used as positive control. Results were expressed as IC50 in µg/mL, with concentration required to reduce the 50% of the initial lipoxigenase activity.

### 2.6. Statistical Analysis

All data was recorded as mean ± standard deviation of triplicate determinations. The statistical analysis was carried out by analysis of variance (ANOVA), followed by Tukey’s test using the IBM-SPSS Statistics software, version V15 (New York, NY, USA). The significance level was set at *p* < 0.05. Principal component analysis (PCA) and cluster analysis were performed using Minitab 17 statistical software (Minitab, LLC, State College, PA USA).

## 3. Results and Discussion

### 3.1. Pressurized Liquid Extraction of Ammodaucus leucotrichus

**Figure 1:**
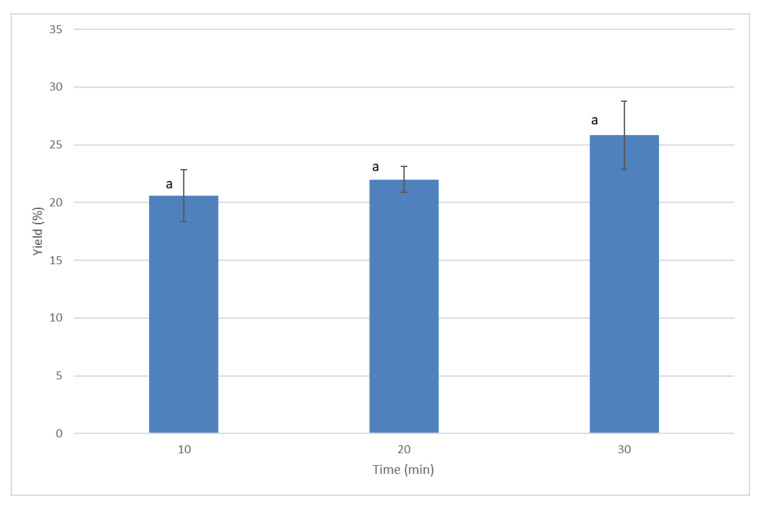
Extraction recoveries of *Ammodaucus leucotrichus* by pressurized ethanol at 110 °C and 1500 Psi (10.34 MPa) at different extraction times. Mean values with different superscript letters are significantly different (*p* < 0.05).

In order to establish the extraction time, the extraction yield was studied at 10, 20 and 30 min using pressurized ethanol at 110 °C and 1500 Psi (10.34 MPa). According to the statistical analysis and as illustrated in Figure 1, there were no significant differences between extraction yield after the prolonged time of extraction. For this reason, 10 min was considered as adequate extraction time in these conditions, in order to avoid unwanted reactions due to longer extraction times, as well as to save energy in the extraction process.

**Figure 2:**
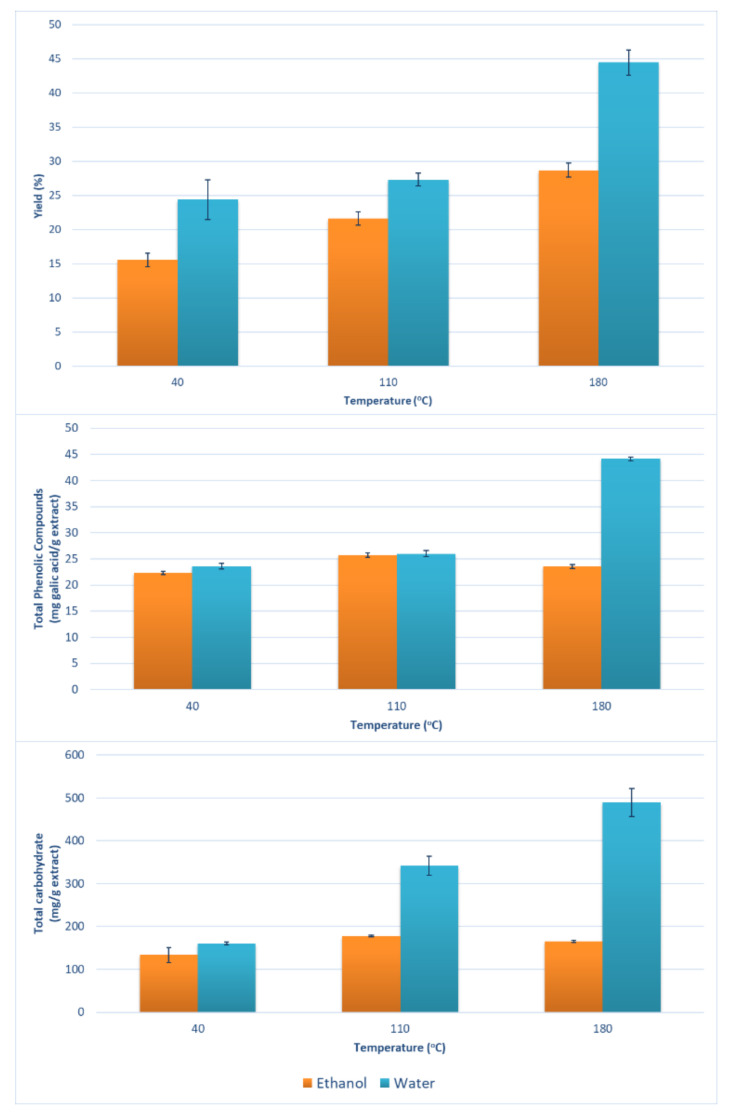
Extraction yield, total Phenolics (mg GAE/g extract) and total carbohydrates (mg/g extract) determined in the extracts of *Ammodaucus leucotrichus* obtained using 10 min pressurized liquid extraction at the indicated conditions.

Water and ethanol due to their low environmental impacts were used as extraction solvents at different temperatures, to extract bioactive molecules from *Ammodaucus leucotrichus* fruits. The results were reported in Figure 2 and Table S1.

As expected, despite the solvent, the extraction yield increased significantly with temperature. Moreover, the most significant increase of extraction yield with temperature was observed when using water at 180 °C (see Figure 2). However, the highest extraction yield obtained reached 44.44%–this value is three times higher than the lowest value (15.55%), which was obtained at 40 °C using EtOH. The use of ethanol led to a lower yield compared with water. This behavior of increasing yield with temperature may also be explained by enhanced mass transfer properties, hence better solubilities at higher temperature. These results are in agreements with those reported in [16,28].

The highest total phenolic content (43.5 ± 0.8 mg GAE/g extract) and total carbohydrate content (489.36 ± 6.64 mg/g extract) were observed at 180 °C using water (the same conditions leading to the highest yield). On the other hand, the use of EtOH at 40 °C gave the lowest values of total phenolic and total carbohydrate contents were 22.3 ± 0.3 mg GAE/g extract and 133.63 ± 13.42 mg/g extract, respectively. Total phenolic and total carbohydrate contents were positively influenced by the temperature when using water. In contrast, when ethanol was used, no significant effect of temperature on total phenols or the total carbohydrate content was observed.

Previous research by Ziania et al. [10] also described high amount of carbohydrates, mainly soluble sugars such as glucose, fructose and sucrose, in the hydroethanolic extracts of *Ammodaucus leucotrichus* of the order of 65 g/100g dw (equivalent to 650 mg/g extract), comparatively to *Moringa oleifera* with 56.6 g/100g dw (equivalent to 566 mg/g extract), considering this plant as a source of high energetic value.

The high values found with water at 180 °C may be due to the higher solubility of compounds at higher temperature, as well as a possible complex protein and carbohydrates degradation to smaller compounds that could have been easily extracted within the 10 min extraction time. Furthermore, small peptides can be detected using the Folin-Ciocalteau test. In addition, starch hydrolysis using pressurized hot water has been proposed and successfully used by previous researchers [35,36].

### 3.2. In Vitro Assays

Table 1 shows the results for anti-cholinesterase (AChE), anti-inflammatory (LOX) and antioxidant capacity (DPPH radical scavenging) of the *Ammodaucus leucotrichus* extracts. All the results are expressed as IC50 (μg/mL), which means that higher activities are achieved with lower IC50 values. In this sense, the extract obtained using water at 180 °C provided the best values for all of them.

Regarding the neuroprotective activity measured by the acetylcholinesterase inhibition assay, it is clear from the data of Table 1 that 180 °C provided the highest values of inhibition for both ethanol and water solvents. In fact, at lower temperatures it was not possible to determine IC50 for ethanol extracts. The best values obtained with water indicate the higher polarity of compounds responsible for this activity, whose composition will be seen later. Following the steepest ascent of AChE with temperature; 200 °C was tested using pressurized water to confirm whether the combined effect of higher temperature could improve AChE activity. However, the IC50 value obtained at 200 °C was higher to that obtained at 180 °C, with 189.430 μg/mL at 200 °C vs. 55.598 μg/mL at 180 °C, respectively. This demonstrated that it was not necessary to increase the temperature to the maximum one tested initially. Previous researchers found relations between the biological activity of extracts obtained from natural matrices at a high temperature using water with the formation of new compounds from Maillard and caramelization reactions [37,38,39]. In fact, the appearance of brown color, which we found at 200 °C with just a visual estimation, indicate the presence of Maillard reaction products.

Sadaouia et al. studied the inhibitory potential against acetylcholinesterase of the *A. leucotrichus* aerial parts essential oil, and they could not achieve an IC50 value for the AChE activity in their extracts–they only provided value for certain pure compounds present in the extract [11]. Therefore, in the present paper, and to the best of the authors’ knowledge, it was the first time the AChE inhibitory activity of *A. leucotrichus* extracts was measured. On the contrary, the antioxidant capacity measured by DPPH radical scavenging capacity did not show the same effect with the solvent, but rather showed a dependency on temperature (higher temperature, higher activity). This trend is similar to the one found in the TPC assay, where in fact, the reaction of reduction of molibdotungstate (Folin reagent) is another way to express antioxidant activity. It is common to find the same trend in both values when the main antioxidant compounds present in the sample are phenolic compounds, as will be seen in the following section.

This last effect was also seen in the anti-inflammatory activity measured by lipoxygenase inhibition test (LOX), but better values were found in ethanol, except for the extract obtained with water at 180 °C, which provided again the best value. In fact, no significant difference was observed between the extract obtained at 180 °C using water and quercetin, which was used as a positive control. Again, to date, neither the inhibition of lipoxygenase nor other anti-inflammatory activity of *Ammodaucus leucotrichus* have ever been studied.

The best results found for in vitro activities for water at 180 °C were in full agreement with the highest values of total phenolic and total carbohydrate contents, in the same conditions (180 °C and water); even then, it was the extraction condition that provided the best yield. Their composition will be seen in the following section.

In summary, these findings put into evidence the potential of *A. leucotrichus* extracts as inhibitors of AChE, LOX and antioxidants. Thus, the most interesting multibioactive extracts of AL would be those extracted with water at a high temperature.

### 3.3. Chemical Characterizations

**Figure 3:**
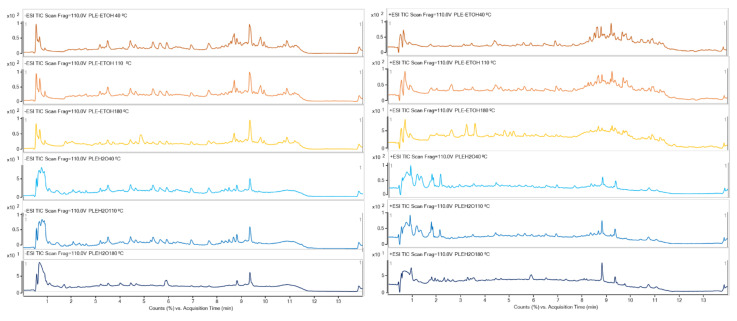
UHPL-ESI-qTOF Chromatograms (Total Ionic Current, TIC) of the pressurized liquid extracts of *Ammodaucus leucotrichus* fruits obtained using 10 min of extraction time at indicated temperatures. Left side negative polarity, right side positive polarity. Orange chromatograms correspond to ethanolic extracts, blue chromatograms correspond to water extracts. Extraction temperature is indicated above each chromatogram.

One of the aims of this study was to determine the profile of the compounds present in *Ammodaucus leucotrichus* fruit extract. Thus, the untargeted analysis of all the extracts were carried out by UHPLC-q-TOF-MS/MS. It can be clearly seen in Figure 3 and Table S2 that the qualitative profile varied depending on the considered sample.

**Figure 4:**
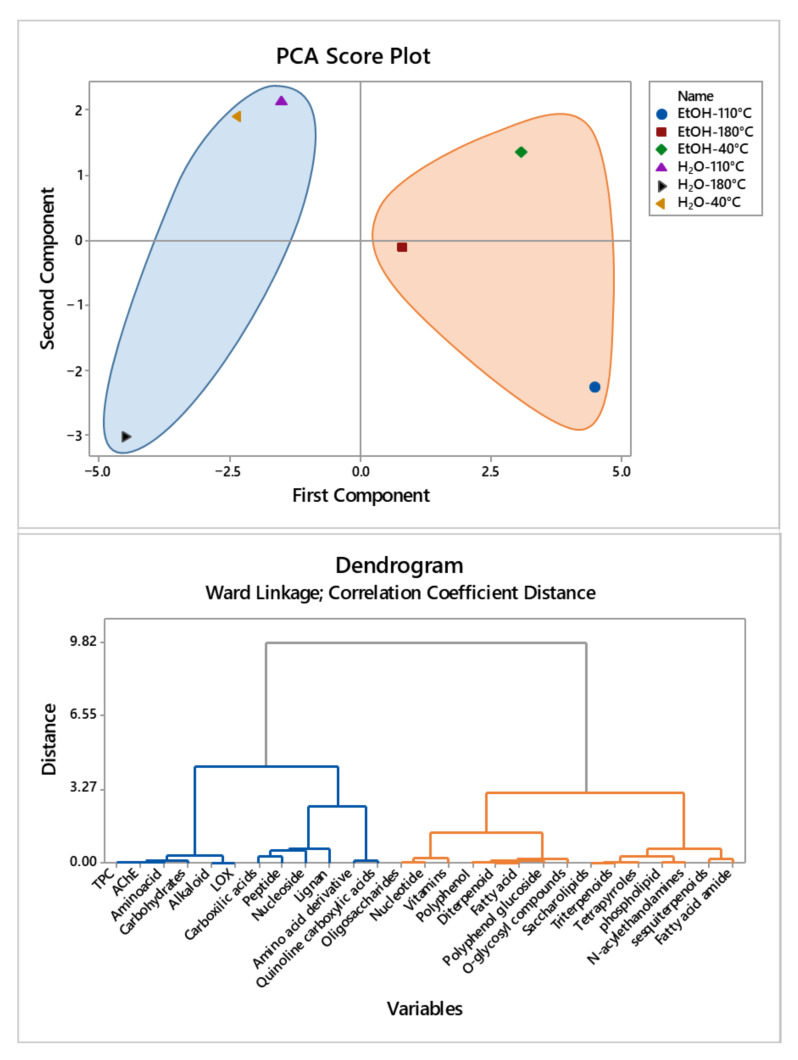
Multivariate analyses of *Ammodaucus leucotrichus* extracts. Score plot obtained in Principal Component Analysis (PCA), and below, a dendogram combining bioactivities and composition of extracts classified by families obtained. Note: Principal component 1 and 2 are the first two components from PCA that capture 67.3% of variation.

Overall, 94 compounds were tentatively identified, mainly phenolic compounds free and glycosylated, as well as lipids and organic acids. Since there are few reports that could be found in the literature on the phytochemical analysis of *A. leucotrichus* fruit composition extract, it is difficult to compare with the results obtained in the present work. Ziania et al. [10] studied aerial parts composition of *A. leucotrichus*, and they confirmed the presence of some phenolic compounds such as Apigenin-6,8-C-diglucoside, Luteolin-7-O-glucoside, Di-O-caffeoyl-malonylquinic acid, Luteolin-O-(malonyl-hexoside) isomer and Di-O-caffeoyl-dimalonylquinic in the ethanolic extract (80% *v/v*) of *A. leucotrichus* fruit. Therefore, the detected presence of the Flavonoid derivatives in *Ammodaucus leucotrichus* fruit extract was not surprising [10]. Different studies have shown that the Flavonoid glycosides possess a wide variety of pharmacological activities, such as antioxidant and anti-inflammatory [40,41]. Other compounds identification was based on the MS data; only some of them could be confirmed by previous research in this plant or taxonomical members [42,43,44,45,46,47,48,49,50,51]. The bioactivity cannot be assigned by a single compound, but in synergy between a number of compounds. In order to find correlations among the composition and the obtained bioactivities (AChE and LOX), multivariate statistical analysis (combining Principal Component Analysis (PCA) and cluster analysis) was performed using Minitab statistical software. Despite 94 individual compounds being detected, not all of them were present in all the samples, so these correlation studies were carried out considering families of compounds using the normalized areas. Furthermore, taking into account that the best activities provide lower IC50 values, the values used are the inverse of those of Table S2. The graphical results of these multivariate analyses can be seen in Figure 4.

The PCA allowed the classification in two main groups using only two components, which could capture 67.3% of the encountered experimental variations. With four components, the total variability of the data was accounted for. It can be easily seen that the first component PC1 can classify extracts regarding the extraction solvent obtaining negative values of PC1 for water (blue region) and positive PC1 values for ethanol extractions (orange region). Regarding the second component of the multivariate analysis (PC2), it allowed to classify the extracts regarding their anti-inflammatory capacity measured by LOX, providing negative PC2 values to those samples with higher potential. The main point of this analysis was the possibility to discriminate the sample with higher bioactivity (water 180 °C) in terms of being anti-inflammatory (LOX) and antineurodegenerative (AChE)–this sample is the only one whose values of PC1 and PC2 are negatives.

The cluster analysis results were similar. Two main clusters were obtained, which can be seen in Figure 4 (lower graph). On the left, the blue cluster gathers together the fractions with higher correlation with bioactivity, as well as high amounts of total carbohydrates and phenolics compounds, and free amino acids, peptides and other compounds. This composition can be found in the *Ammodaucus leucotrichus* extracts obtained with water at 180 °C.

## 4. Conclusions

Pressurized liquid extraction using green solvents has been developed for the first time to extract bioactive molecules from *Ammodaucus leucotrichus*. It can be regarded as a first step in the valorization of this underexplored and unexploited North African natural plant potential.

In fact, the developed method consisting of PLE using water can be considered as a green technique to extract bioactive molecules without organic solvent in only 10 min.

The results indicated that the highest yield was 44.44%, and total phenolics content and total carbohydrate content were achieved in the same condition, at 180 °C using water. Moreover, under the same conditions, the extracts obtained presented a remarkable AChE (IC50 = 55.598 μg/mL) and LOX (IC50 = 39.373 μg/mL) inhibition, as well as a good antioxidant capacity. The chemical characterization of those extracts, performed by the UHPLC-q-TOF-MS/MS, allowed the identification of a wide variety of secondary metabolites from *Ammodauus leucotrichus* fruits, and the multivariate analysis allowed us to correlate the bioactivity with the compositions of phenolic compounds and carbohydrates, which are the main compounds associated with high anti-inflammatory and neuroprotective activities.

It can be concluded that water is an ideal solvent in terms of extraction recovery, also providing an attractive bioactivity potential against neurodegenerative diseases. However, these interesting results encourage further works on the biological effect of the *Ammodaucus leucotrichus* plant. Moreover, this study showed a worthwhile bioactivity potential for future pharmaceutical and food applications. In vivo studies should be carried out to confirm the potential described in the present article.

## Figures and Tables

**Table 1:** IC50 (μg/mL) values from in vitro assays of different *Ammodaucus leucotrichus* extracts using AChE, LOX, DPPH assays.

Samples	AChE	LOX	DPPH
EtOH-40 °C	n.d	197.621 ± 5.646 ^f^	287.699 ± 1.816 ^f^
EtOH-110 °C	n.d	140.076 ± 9.076 ^e^	92.200 ± 6.067 ^c^
EtOH-180 °C	300.458 ± 18.275 ^d^	107.973 ± 14.001 ^e^	60.482 ± 0.176 ^b^
H_2_O-40 °C	316.817 ± 36.329 ^d^	536.985 ± 7.255 ^d^	129.711 ± 7.146 ^e^
H_2_O-110 °C	222.329 ± 32.459 ^c^	342.311 ± 5.510 ^c^	111.184 ± 4.176 ^d^
H_2_O-180 °C	55.598 ± 7.724 ^b^	39.373 ± 4.783 ^b^	58.513 ± 4.756 ^b^
Positive control *	4.061 ± 0.310 ^a^	14.298 ± 1.748 ^a^	18.714 ± 1.301 ^a^

n.d: not determined (maximum level of inhibition below 50%). The values are means ±sd. Different superscripts (a, b, c, d, e, f) indicate significant differences (*p* ≤ 0.05). * Chemical standards used as positive controls of each test: Galantamine for AChE, Quercetin for LOX and BHT for DPPH.

## Data Availability

Not applicable.

## Supplementary Materials

Figure S1. Representative pictures of Ammodaucus leucotrichus fruit; ground material (left), raw material (right). Table S1. Extraction yield, total Phenolics (mg GAE/g extract) and total carbohydrates (mg/g extract) determined in the pressurized liquid extracts of *Ammodaucus leucotrichus* fruits obtained using 10 min of extraction time at the indicated conditions. Table S2. Identification and mean areas found in the UHPL-ESI-qTOF (positive and negative polarity) Chromatograms of the pressurized liquid extracts of Ammodaucus leucotrichus fruits obtained using 10 min of extraction time at indicated temperatures and solvents.

## Sample Availability

Samples of the compounds are not available from the authors.

## Abbreviations

ABD-F, 4-Fluoro-7-sulfamoyl benzofurazan; Ach, acetylcholine; AChE, acetylcholinesterase; ACth, Acetylthiocholine iodide; AL, *Ammodaucus leucotrichus*; ANOVA, analysis of variance; BHT, Butylated Hydroxytoluene; DI, degree of inhibition; DMSO, dimethyl sulfoxide; DPPH, 2,2-diphenyl-1-picrylhydrazyl; DW, dry weight; EtOH, ethanol; GAE, Gallic acid equivalence; GNPS, global natural products social molecular networking; GRAS, generally recognized safe; H2O, water; IC50, inhibitory concentration at 50%; LA, linoleic acid; LC, liquid chromatog-raphy; LOX, lipoxidase; PCA, principle components analysis; PLE, pressurized liquid extraction; q-TOF-MS/MS, quadrupole time-of-flight tandem mass spectroscopy; TPC, total phenolic compound; TC, total carbohydrate; ε, dielectric constant.
