# Supplementary material for: Optimization of Pressurized Liquid Extraction and In Vitro Neuroprotective Evaluation of Ammodaucus leucotrichus. Untargeted Metabolomics Analysis by UHPLC-MS/MS
Source: Molecules. 2021 Nov 17;26(22):6951. doi: 10.3390/molecules26226951 (PMC8625519; doi:10.3390/molecules26226951)
Supplement: Supplementary file 1 [file molecules-26-06951-s001.zip › molecules-1464706-supplementary.pdf]

Supplementary material of: Optimization of pressurized liquid extraction and in vitro neuroprotective evaluation of *Ammodaucus leucotrichus*. Untargeted Metabolomics Analysis by UHPLC-MS/MS

Norelhouda Abderrezag[1,2], José David Sánchez-Martínez[2], Ouahida Louaer[1], Abdeslam-Hassen Meniai[1] and Jose A. Mendiola [2,*]

1 Laboratory of Environmental Process Engineering, Faculty of Process Engineering, University Salah Boubnider – Constantine 3, 25000 Constantine, Algeria.
2 Foodomics Laboratory, Bioactivity and Food Analysis Department, Institute of Food Science Research CIAL (CSIC-UAM), C/ Nicolás Cabrera 9, 28049 Madrid, Spain
* Correspondence: j.mendiola@csic.es

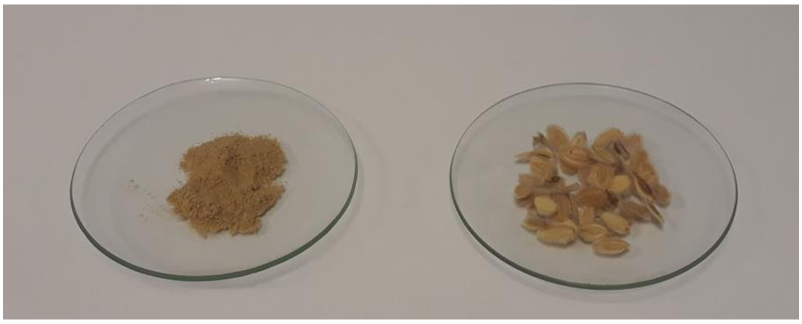

Figure S1. Representative pictures of *Ammodaucus leucotrichus* fruit; ground material (left), raw material (right).

Table S1. Extraction yield, total Phenolics (mg GAE/g extract) and total carbohydrates (mg/g extract) determined in the pressurized liquid extracts of *Ammodaucus leucotrichus* fruits obtained using 10 min of extraction time at the indicated conditions.

Solvent	Temperature (°C)	Total yield (% , w/w)	Total Phenolics (mg GAE/g Extract)	Total Carbohydrates (mg/g Extract)
Ethanol	40	15.55 ± 0,89[a]	22.3 ± 0.3[a]	133.63 ± 13.42[a]
	110	21.61 ± 1.70[b]	25.8 ± 0.4[c]	177.52 ± 1.24[a]
	180	28.71 ± 0.36[d]	23.6 ± 0.4[b]	165.02 ± 1.60[a]
Water	40	24.38 ± 0.70[c]	23.6 ± 0.5[b]	160.34 ± 2.18[a]
	110	27.25 ± 0.25[d]	26.0 ± 0.5[c]	342.12 ± 6.39[b]
	180	44.44 ± 0.82[e]	43.5 ± 0.8[d]	489.36 ± 6.64[c]

Note: Each data point represents the mean ± SD of replicates. Different superscript letters indicate statistically significant differences ($p < 0.05$) per column

Table S2. Identification and mean areas found in the UHPLC-ESI-qTOF Chromatograms of the pressurized liquid extracts of *Ammodaucus leucotrichus* fruits obtained using 10 min of extraction time at indicated temperatures and solvents. [M+H]+ indicate positive ESI polarity, [M-H]- indicate negative ESI polarity

#	Rt (min)	Tentative identification	Molecular formula	Monoisotopic mass	[M+H]+	[M-H]-	Area counts						Ref
							H_2O-180°C	H_2O-110°C	H_2O -40°C	ETOH-180°C	ETOH-110°C	ETOH-40°C	
**1**	0,547	D-mannitol	C6H14O6	182.079	183.0866	/	-	-	-	256913.06	-	160672.51	[42]
**2**	0,592	Citric acid	C6H8O7	192.027	/	191.0223	353378.81	195974.47	4210281.72	-	-	-	[43]
**3**	0,629	Melezitose	C18H32O16	504.169	522.2039	/	-	-	-	29584.98	27162.81	28271.3	
**4**	0,634	Isomaltulose	C12H22O11	342.1162	360.1487	/	-	-	-	-	-	135561.23	
**5**	0,666	Trehalose	C12H22O11	342.1162	/	341.1067	-	-	-	-	-	32729.68	
**6**	0,69	N-fructosyl pyroglutamate	C11H17NO8	291.0954	/	290.0859	-	-	34060.24	299069.54	826707.56	382906.83	
**7**	0,707	Adenine	C5H5N5	135.0544	136.0615	/	-	-	-	46760.03	-	-	
**8**	0,727	5-deoxy-5-(methylsulfinyl)adenosine	C11H15N5O4S	313.0844	314.0934	/	35673.29	120821.03	112437.96	27709.55	-	75402.01	
**9**	0,73	Adenosine	C10H13N5O4	267.0967	268.1035	/	-	-	-	383507.74	-	121233.07	
**10**	0,759	N-fructosyl isoleucine	C12H23NO7	293.1474	294.153	/	-	-	-	-	-	67835.07	
**11**	0,768	Adenine hydrochloride	C5H6ClN5	171.0311	136.0607	/	-	-	-	-	2073.85	95037.61	
**12**	1,089	His-pro l-histidyl-l-proline	C11H16N4O3	252.1222	235.1213	/	63766.14	-	-	-	-	-	
**13**	1,125	5-methylcytosine	C5H7N3O	125.0589	126.0669	/	-	231184.42	-	-	-	-	
**14**	1,139	Isoleucine	C6H13NO2	131.0946	132.101	/	625928.34	-	-	-	-	-	[43]
**15**	1,329	Meglutol (aka 3-hydroxymethylglutaric acid)	C6H10O5	162.0528	/	161.0447	-	1271229.98	-	-	-	-	
**16**	1,698	Phe-arg	C15H23N5O3	321.1800	322.1921	/	-	-	44413.58	-	-	-	
**17**	1,774	α-adenosine	C10H13N5O4	267.0967	268.1077	/	-	-	1136956.03	-	-	-	
**18**	1,832	2-deoxyadenosine	C10H13N5O3	251.1018	252.1112	/	-	754326.63	-	-	-	-	
**19**	1,892	Guanosine	C10H13N5O5	283.0916	/	282.0869	-	607330.63	525654.93		22931.34	-	
**20**	2,174	Leu-leu-arg l-leucyl-l-leucyl-l-arginine	C18H36N6O4	400.2798	401.2916	/	-	-	20611.77	-	-	-	
**21**	2,178	2`-o-methylguanosine	C11H15N5O5	297.1073	298.1144	/	-	16989.46	-	-	-	-	
**22**	2,408	Pantothenic acid	C9H17NO5	219.1106	/	218.1017	-	-	-	-	-	40563.84	
**23**	2,479	Chlorogenic acid	C16H18O9	354.0950	/	353.0908	-	26798.15	-	-	-	-	[44]
**24**	2,617	Succinoadenosine	C14H17N5O8	383.1077	384.1193	/	-	42590.91	32102.49	-	-	-	

#	Rt (min)	Tentative identification	Molecular formula	Monoiso-topic mass	[M+H]+	[M-H]-	Area counts						Ref
							H_2O-180°C	H_2O-110°C	H_2O -40°C	ETOH-180°C	ETOH-110°C	ETOH-40°C	
**25**	2,646	N2_n2-dimethylguanosine	C12H17N5O5	311.1229	312.1334	/	-	30971.86	-	-	-	-	
**26**	2,881	Tryptophan	C11H12N2O2	204.0898	205.0976	/	-	170148.31	233265.5	-	-	36387.87	[45]
**27**	2,901	Abrine	C12H14N2O2	218.1055	188.0734	/	-	-	82874.54	-	-	-	
**28**	3,044	Xanthurenic acid	C10H7NO4	205.0375	206.0457	/	-	9935	-	-	-	-	
**29**	3,152	Salidroside	C14H20O7	300.1209	318.1544	/	-	-	-	-	3928.57	14741.76	
**30**	3,696	Olivil 4-o-glucoside	C26H34O12	538.205	556.238	/	-	-	13104.4	29531.1	25746.5	12247.41	[46]
**31**	3,735	Oleuropein	C25H32O13	540.1843	/	537.202	5069.04	-	-	-	-	-	
**32**	3,78	Magnolioside	C16H18O9	354.0950	355.1068	/	-	9854.92	-	-	-	-	
**33**	3,948	Licoagroside b	C18H24O12	432.1267	433.1344	/	-	-	-	-	89624.35	-	
**34**	4,107	Icariside f2	C18H26O10	402.1526	420.1873	/	-	-	-	-	94678.37	-	
**35**	4,115	Norharmane	C11H8N2	168.0687	169.0776	/	60800.27	-	-	-	-	-	
**36**	4,321	Leu-phe l-phenylalanine. L-leucyl-	C15H22N2O3	278.1630	279.171	/	-	-	14156.73	-	-	-	
**37**	4,616	(-)-Erythro-anethole glycol 2-glucoside	C16H24O8	344.1471	/	343.1375	-	216892.16	210475.47	-	349268.58	348151.05	
**38**	4,79	Melibiose	C12H22O11	342.1162	/	341.1111	-	-	-	1714.93	-	5932.14	
**39**	4,812	Isomaltulose	C12H22O11	342.1162	/	341.1099	-	-	-	-	10017.86	-	
**40**	4,862	2-phenylethyl 6-o-[(2s,3r,4r)-3,4-dihydroxy-4-(hydroxymethyl)te-trahydro-2-furanyl]-beta-d-glucopyra-noside	C19H28O10	416.1682	434.2057	/	-	14756.56	12440.55	13111.45	30206.48	19273.33	
**41**	5,232	Scopoletin	C10H8O4	192.0422	193.0499	/	-	-	-	-	92930.27	135092.42	[47]
**42**	5,244	Rosiridin	C16H28O7	332.1835	350.2176	/	-	-	-	-	16601.91	8557.74	
**43**	5,433	Secoisolariciresinol diglucoside	C32H46O16	686.2785	/	731.2734	10303.7	15431.41	-	18553.63	17717.47	21842.93	
**44**	5,473	2-(hydroxymethyl)-6-[4-[(2s.3s)-3-(hydroxymethyl)-5-[(e)-3-hydro-xyprop-enyl]-7-methoxy-2.3-dihydro-benzofuran-2-yl]-2-metho-xyphenoxy]oxane-3.4.5-triol	C26H32O11	520.1944	538.23	/	-	50564.3	-	46097.94	108018.97	97312.73	
**45**	5,546	Phe-pro l-phenylalanyl-l-proline	C14H18N2O3	262.1317	245.1287	/	7327.25	-	-	-	-	-	
**46**	5,6	Luteolin-4-o-glucoside	C21H20O11	448.1005	449.1122	/	-	-	174044.54	-	16414.09	673537.14	[10]
**47**	5,613	Luteolin	C15H10O6	286.0477	287.0557	/	13212.81	64365.75	21756.23	-		81465.26	[48]
**48**	5,628	Luteolin-7-o-glucoside	C21H20O11	448.1005	/	447.0951	69065.61	249806.7	80599.32	472705.88	491488.83	709743.1	[10]
**49**	5,789	Artselaeroside a	C19H28O10	416.1682	/	415.1618	-	13343.51	-	-	-	-	

#	Rt (min)	Tentative identification	Molecular formula	Monoiso-topic mass	[M+H]+	[M-H]-	Area counts						Ref
							H_2O-180°C	H_2O-110°C	H_2O -40°C	ETOH-180°C	ETOH-110°C	ETOH-40°C	
**50**	5,895	Secoisolariciresinol	C20H26O6	362.1729	327.1594	/	-	-	133503.9	108794.11	-	-	
**51**	6,22	Apigetrin	C21H20O10	432.1056	433.1127	/	-	-	-	46851.97	75765.67	-	
**52**	6,228	Aloenin	C19H22O10	410.1213	433.1107		-	-	-	-	-	74898.28	[49]
**53**	6,243	Apigenin-7-o-glucoside	C21H20O10	432.1056	/	431.1014	-	50006.98	-	72551.76	107889.58	138117.72	[50]
**54**	6,272	Apigenin	C15H10O5	270.0528	271.0587	/	-	-	-	-	-	5461.85	[48]
**55**	6,411	Kaempferol 3-o-(6''-malonyl-glucoside)	C24H22O14	534.1009	/	533.093	18989.85	175483.87	19501.18	29675.95	38321.12	52268.82	
**56**	6,439	Peonidin 3-galactoside cation	C22H23ClO11	498.0928	463.1305	/	11870.52	107973.97	33700.05	-	159609.31	22946.27	
**57**	6,474	Hispidulin 4'-glucoside	C22H22O11	462.1162	/	461.1059	-	36257.29	-	61747.79	104199.27	112174.81	
**58**	6,583	Kaempferol-o-acetylhexoside	C23H22O12	490.1111	491.1219	/	-	-	-	-	26539.4	-	
**59**	7,013	Malonylgenistin	C24H22O13	518.106	519.1185	/	-	24689.1	-	-	-	-	
**60**	7,096	(2e)-5-(2.3-dimethyl-tricyclo[2.2.1.0~2.6~]hept-3-yl)-2-methyl-2-pentenoic acid	C15H22O2	234.1619	235.1692	/	-	-	17717.68	-	-	-	
**61**	7,118	Atractyligenin (2-o-beta-glucopyra-nosyl-)	C26H40O8	480.2723	481.4027	/	-	-	-	17160.03	-	22946.27	
**62**	7,538	Z-ajoene	C15H24O3	252.1725	235.1681	/	-	-	-	-	-	25738.6	
**63**	7,573	Luteolin	C15H10O6	286.0477	/	285.0402	91524.94	327175.86	651690.56	1057626.37	1337640.84	1276508.69	[48]
**64**	7,715	Kahweol	C20H26O3	314.1882	332.2072	/	-	-	-	-	16518.87	-	
**65**	7,844	6-o-acetylgenistin	C23H22O11	474.1162	475.1237	/	-	-	20071.13	-	-	-	
**66**	8,237	Apigenin	C15H10O5	270.0528	/	269.0461	-	18256.97	40402.51	35777.26	65299.66	99271	[48]
**67**	8,312	10,15-octadecadienoic acid, 9,12,13-trihydroxy-	C18H32O5	328.2249	/	327.2156	-	-	34544.7	-	-	54520.82	
**68**	8,341	Diosmetin	C16H12O6	300.0633	301.0746	/	-	42782.71		42127.41	98510.86	107058.01	[51]
**69**	8,503	9.12.13-trihydroxy0-octadecenoic acid	C18H34O5	330.2406	/	329.2328	32165.95	215421.58	302602.83	101190.5	253994.81	286558.35	
**70**	8,672	Caffeic acid	C9H8O4	180.0422	163.0397	/	-	-	-	-	56612.35	-	[48]
**71**	8,929	Phytosphingosine	C18H39NO3	317.2929	318.3048	/	-	-	-	-	123447.37	-	
**72**	9,118	Vicine	C10H16N4O7	304.1019	304.3039	/	-	-	-	-	15155.3	-	
**73**	9,424	1-palmitoyl-2-linoleoyl pe	C39H74NO8P	715.5152	/	714.5089	114762.55	11800.26			25952.95	65464.86	
**74**	9,674	Coumaroyl glucose (p-)	C18H15O4P	326.0707	327.0754	/	-	-	-	-	28967.57	947.77	
**75**	9,675	Alpha-dimorphecolic acid	C18H32O3	296.2351	/	295.2278	-	94220.93	-	240380.24	363755.92	453266.03	
**76**	9,716	Vernolic acid	C18H32O3	296.2351	279.2357	/	-	-	-	-	52633.25	-	

#	Rt (min)	Tentative identification	Molecular formula	Monoiso-topic mass	[M+H]+	[M-H]-	Area counts						Ref
							H_2O-180°C	H_2O-110°C	H_2O -40°C	ETOH-180°C	ETOH-110°C	ETOH-40°C	
**77**	9,723	Linolenic acid	C18H30O2	278.2245	279.2357	/	-	-	-	37580.27	-	-	[43]
**78**	9,746	1-palmitoyl-2-hydroxy-sn-glycero-3-phosphoethanolamine	C21H44NO7P	453.2855	/	452.2765	-	-	-	-	33150.36	55686.05	
**79**	9,807	1-(9z-octadecenoyl)-sn-glycero-3-phosphoethanolamine	C23H46NO7P	479.3011	/	478.2894	-	-	-	-	-	25958.79	
**80**	9,918	(-)-Isolongifolol	C15H26O	222.1983	205.1984	/	-	-	-	186569.19	192776.99	-	
**81**	10,009	6-paradol	C17H26O3	278.1882	279.1602	/	-	64549.93	87230.6	78706.47	128249.87	160475.23	
**82**	10,069	Lyso-pc(16:0) 1-palmitoyl-sn-glycero-3-phosphocholine	C24H50NO7P	495.3324	496.3432	/	-	-	-	89909.81	-	-	
**83**	10,219	1-(9z-octadecenoyl)-sn-glycero-3-phosphocholine	C26H52NO7P	521.3481	522.3549	/	43610.79	-	-	92824.45	236486.76	-	
**84**	10,233	Linoleoyl ethanolamide	C20H37NO2	323.2824	324.2902	/	-	-	-	36625.55	66053.78	59444.5	
**85**	10,251	Lpc 18:1 1-oleoyl-sn-glycero-3-phosphocholine	C26H52NO7P	521.3481	/	566.3447	-	-	-	-	50191.37	56596.86	
**86**	10,593	16-hydroxypalmitic acid	C16H32O3	272.2351	/	-	-	-	-	-	154253.85	-	
**87**	10,662	N-oleoylethanolamine	C20H39NO2	325.298	326.3026	/	-	-	-	-	46449.9	2167.11	
**88**	10,761	Oleanolic acid	C30H48O3	456.3603	439.3563	/	-	-	-	-	39705.62	-	
**89**	10,831	9-octadecenamide. (z)-	C18H35NO	281.2718	282.2799	/	-	-	-	729370.61	775760.87	597323.34	
**90**	11,083	Pheophorbide a	C35H36N4O5	592.2685	593.2706	/	-	-	-	32435.93	102694.61	-	
**91**	11,633	Dihydroperillic acid glucuronide	C20H24N2O2	326.10016	/	325.1848	1870.22	-	-	-	-	-	
**92**	11,841	Resveratrol 5-o-glucoside	C20H22O8	390.13146	391.2838	/	-	-	-	-	6691.07	-	
**93**	13,074	MGMG (16:3)	C25H42O9	486.282885	531.4067	/	-	-	-	9813.91	11807.11	14406.67	
**94**	13,902	Pi 34:2 phosphatidylinositol(34:2)	C43H79O13P	835.06799	/	833.5184	-	-	-	-	1344.31	956.12	
